# Integrin β3 α2,6-sialylation by the sialyltransferase ST6Gal1 promotes human cytomegalovirus entry into fibroblasts

**DOI:** 10.1080/21505594.2026.2732519

**Published:** 2026-09-11

**Authors:** Taowen Pan, Huiyi Wang, Liyuan Guo, Guogan Tang, Luping Zheng

**Affiliations:** a Institute (College) of Integrative Medicine, Dalian Medical University, Dalian, China; b The First Affiliated Hospital of Dalian Medical University, Dalian, China

**Keywords:** Human cytomegalovirus, Integrin β3, α2,6-sialylation, ST6Gal1, viral entry

## Abstract

The interactions between viral ligands and cellular receptors, along with the subsequent entry of Human cytomegalovirus (HCMV) into host cells, represent an important focus of research in virus-host relationships. This study investigated the function and mechanistic role of α2,6-sialylation of Integrin β3 during the early stages of infection in fibroblasts. Analysis of the human Integrin β3 glycomics database revealed that the surface glycans of Integrin β3 are extensively modified with terminal sialic acid residues and occur at relatively high abundance. To evaluate their functional relevance, Integrin β3-knockout cells and α2,6-sialyltransferase ST6Gal1-knockdown cells were established. Quantitative PCR, flow cytometry, and immunofluorescence analyses confirmed that α2,6-sialylation of Integrin β3 is required for viral internalization, and the absence of this modification significantly reduced viral entry into host cells. This observation was further validated through additional virological assays designed to detect viral infection. Mechanistic analysis demonstrated that defective α2,6-sialylation of Integrin β3 impairs the interaction between viral receptors and ligands, preventing activation of the viral entry signaling pathways, Integrin-Src, and resulting in impaired HCMV-induced Rho A-cofilin inactivation. These findings broaden the current understanding of early HCMV infection and highlight ST6Gal1 and Integrin β3 α2,6-sialylation as potential glycobiological targets for therapeutic intervention.

## Introduction

Human cytomegalovirus (HCMV) is classified as a β-herpesvirus containing a 235 kb double-stranded DNA genome. Global seroprevalence of HCMV varies according to socioeconomic factors, with higher prevalence among individuals from lower socioeconomic backgrounds. The estimated average global seroprevalence is approximately 83% [1]. HCMV infection produces a broad spectrum of clinical outcomes, ranging from asymptomatic infection in healthy hosts to severe or fatal disease in immunocompromised individuals, including transplant recipients, patients with AIDS, and immunologically immature neonates [2,3]. Current therapeutic options for HCMV are limited by suboptimal efficacy and dose-limiting toxicities [4,5]. Therefore, a deeper understanding of HCMV pathogenesis and the development of novel antiviral strategies remain critical research priorities.

HCMV displays broad cellular tropism, infecting fibroblasts, epithelial and endothelial cells, and macrophages [6,7]. This tropism is regulated by the selective use of viral ligands on the virion. The trimer gH/gL/gO facilitates infection of fibroblasts, whereas the pentamer gH/gL/UL128/UL130/UL131 enables infection of epithelial and endothelial cells, leading to gB-mediated membrane fusion [8,9]. In fibroblasts, HCMV primarily establishes a lytic infection. Early infection involves virus attachment and internalization. Following initial binding to nonspecific receptors such as heparan sulfate proteoglycans (HSPGs) [10], the virus engages specific receptors, with multiple viral glycoproteins coordinating membrane fusion and entry. Key viral receptors mediating fibroblast infection include Integrin β3, epidermal growth factor receptor (EGFR), and platelet-derived growth factor receptor α (PDGFRα) [11–13]. Integrin β3 is a well-characterized receptor for HCMV entry and interacts with viral glycoprotein trimers to activate downstream signaling pathways, including Integrin β3-Src and RhoA-cofilin signaling [14], driving cytoskeletal rearrangements that facilitate viral entry.

The glycan composition of viral receptors, characterized by specific monosaccharide linkages at branched or terminal positions, plays a crucial role in early viral infection by modulating protein conformation. Viruses utilize these glycan motifs to enhance receptor binding, facilitating viral attachment and internalization. For example, the hemagglutinin of human influenza virus preferentially binds α2,6-linked sialic acid (SA) on respiratory epithelial cells, whereas the hemagglutinin of avian influenza virus preferentially binds α2,3-linked SA on host cells [15]. In the case of norovirus, histo-blood group antigens (HBGAs) on intestinal epithelial cells serve as receptors, and α1,2-fucosylation of HBGA precursors, catalyzed by the FUT2 gene, enhances host susceptibility to infection [16]. Similarly, transmembrane syndecan-1 (SDC1), a member of the HSPG core protein family, serves as a primary attachment receptor for hepatitis C virus (HCV) [17]. These findings indicate that specific glycan structures or modifications of viral receptors may represent potential targets for blocking early viral infection.

ST6Gal1, an α2,6-sialyltransferase, is the primary glycosyltransferase responsible for catalyzing α2,6-sialylation of target molecules. It is mainly localized in the trans-Golgi and is highly expressed in hepatocytes, B lymphocytes, and intestinal epithelial cells. ST6Gal1 recognizes terminal Galβ1-4GlcNAc motifs on acceptor glycans and uses cytidine monophosphate (CMP)-sialic acid as the donor to transfer sialic acid to the C6 hydroxyl group of galactose. This reaction proceeds through an inverting mechanism mediated by active-site residues, generating an α2,6-glycosidic linkage and releasing CMP [18].

Previous studies have reported that mutations at both N- and O-glycosylation sites of Integrin β3 are associated with HCMV internalization in fibroblasts [18,19]. However, the specific contribution of glycan structures present on the Integrin β3 surface during viral entry remains unclear. In this study, the glycosylation patterns of Integrin β3 were characterized with respect to glycan composition and relative abundance, and the role of α2,6-linked SA on the Integrin β3 surface during early HCMV infection was assessed. The results demonstrate that Integrin β3 α2,6-sialylation catalyzed by ST6Gal1 is involved in HCMV internalization into fibroblasts rather than viral attachment, likely by enhancing interactions between viral entry ligands and Integrin β3. These results expand the current understanding of the mechanisms underlying HCMV infection and highlight potential glycan targets for the development of antiviral strategies.

## Materials and methods

### Cell culture

MRC-5 human lung fibroblasts (ATCC, CCL-171) and HEK 2 lines were incubated at2 mM GlutaMax. HEK 293T cells were cultured in Dulbecco’s Modified Eagle Medium (DMEM) containing 10% FBS. All cell lines were incubated at 37°C in a humidified atmosphere with 5% CO_2_.

### Integrin β3 glycomic analysis

Groups of glycan structures associated with Integrin β3 and their relative abundance at N- and O-glycosylation sites were analyzed using high-resolution LC-MS/MS datasets. The datasets correspond to the iProX identifiers PXD060540 and PXD061775 deposited in the ProteomeXchange Consortium and previously submitted by the authors [19,20].

### Glycosidase pretreatment

Recombinant His-tagged Integrin β3 was expressed and purified as previously described [19,20]. Purified protein samples were incubated with Neuraminidase (New England BioLabs, Cat#P0720S, Ipswich, USA) together with the corresponding 1× GlycoBuffer. Enzymatic digestion was performed according to the manufacturer’s protocol. Following digestion, buffer exchange was performed with a 30 kDa centrifugal filter unit (Millipore, USA) to remove residual enzymes and salts. The samples were subsequently resuspended in phosphate-buffered saline (PBS, pH 7.4) for downstream experiments. The degree of recombinant Integrin β3 de-glycosylation was assessed by detecting the mobility shifts using Western blot analysis and *Sambucus nigra* (SNA) lectin blot.

### ST6Gal I gene knockdown and overexpression

Two pre-designed small hairpin RNAs (shRNAs) targeting human ST6Gal1 mRNA (shRNA1 and shRNA2; sequences listed in Table S1) were evaluated for knockdown efficiency. Based on its superior silencing activity, shRNA1 was selected for subsequent experiments (Figure S2(A)). Wild-type Integrin β3 plasmid [19] and the selected ST6Gal1 shRNA vector were co-transfected into HEK 293T or Integrin β3 KO MRC-5 cells using the Lipofectamine® 3000 transfection kit according to the manufacturer’s instructions to obtain α2,6-sialylation-defective Integrin β3 recombinants or ST6Gal1 gene knockdown MRC-5 cells, respectively. For ST6Gal1 overexpression, the ST6Gal1 expression vector (Genepharma Co., Ltd, Shanghai, China) was transfected into MRC-5 cells using the same transfection protocol to upregulate ST6Gal1 expression. Knockdown and overexpression efficiencies were validated by RT-PCR and Western analysis (primers listed in Table S2). Expression of α2,6-SA was assessed using SNA lectin blot.

### SNA lectin blot

Equal amounts of total cell lysates or purified integrin β3 from each group were subjected to SDS-PAGE and transferred onto PVDF membranes. Following blocking with 1× Carbo-Free Blocking Solution (Vector, Cat#SP-5040–125, Burlingame, USA), the membranes were incubated with 10 μg/mL biotinylated SNA (Vector, Cat#B-1305–2, Newark, USA) for 45 min at room temperature. The membranes were then washed three times with TPBS for 10 min each. HRP-conjugated streptavidin was used to detect the bound biotinylated lectin, and the blots were visualized by ECL. Coomassie brilliant blue staining of the gel was used for normalization.

### Viral infection detection

The Towne strain of HCMV (ATCC, VR977) was amplified under standard conditions, and viral titers were determined in MRC-5 cells following the reported procedures [21]. A virus inoculum at an MOI of 1 was added to cells for 2 h at 37°C unless otherwise stated. For experimental optimization, specific assays were performed using viral inocula at different MOIs. For infection experiments, cells from each group were plated and exposed to HCMV under the indicated conditions. At 72 h post-infection (hpi), cellular morphology and virus-induced cytopathic effects (CPE) were assessed while employing light microscopy. Viral genomic DNA was isolated at 48 hpi, and quantitative real-time PCR (qPCR) was conducted to quantify viral DNA copy numbers. Expression of HCMV immediate-early (IE) proteins was analyzed at 48 hpi using Western blotting or immunofluorescence staining. Similarly, progeny virus production was evaluated by determining the median tissue culture infectious dose (TCID_50_) at 3, 5, and 7 days post-infection (dpi) using the Reed–Muench method [22].

### Viral attachment and internalization assay

For viral attachment, cells were incubated with HCMV in fresh medium containing 0.1% pre-cooled paraformaldehyde at 4°C for 1 h. For internalization assays, cells were first incubated with virus at 4°C for 1 h, then at 37°C for 4 h. Surface-bound virus was removed by treatment with trypsin (100 μg/mL) for 3 min at 37°C.

### qPCR for HCMV attachment and internalization

DNA was isolated using the TIANamp Genomic DNA Kit (TIANGEN, Cat#4992254, Beijing, China) according to the manufacturer’s instructions. qPCR was carried out using Taq Pro Universal SYBR qPCR Master Mix (Vazyme, Cat#Q712-02, Nanjing, China) on a Real-Time PCR Detection System in a final volume of 20 μL containing 10 ng of total DNA. Primer pairs specific for the HCMV UL123 gene are given in Table S3. The amplification protocol included an initial denaturation step at 95°C for 3 min, followed by 40 cycles consisting of 95°C for 5 s and 60°C for 30 s. Absolute viral DNA copy numbers were determined based on a standard curve generated from serial dilutions of quantified HCMV 169 strain DNA (Advanced Biotechnologies, Cat#08–925-000, Columbia, USA) [19,23].

### Flow cytometry analysis of HCMV attachment and internalization

Flow cytometry was performed as previously described [19]. Briefly, cells for HCMV binding and internalization were gently detached using a Corning Cellstripper and washed with PBS. Fixation was performed with 3% paraformaldehyde for 5 min, followed by permeabilization in 90% methanol for 10 min on ice. Cells were stained with anti-HCMV pp28 monoclonal antibody (1: 500) (Virusys, Cat#CA004, Taneytown, USA) or mouse isotype control (BD Biosciences, Cat#554126, San Jose, USA) for 60 min, followed by FITC-conjugated anti-mouse IgG (1: 50) (BD Biosciences, Cat#562028, San Jose, USA) for 30 min. Samples were analyzed using a BD FACSverse cytometer, and data were processed with FlowJo software (Tree-Star Inc., Ashland, OR). A minimum of 10^4^ cells were collected and analyzed for each sample.

### Detection of HCMV attachment and internalization by immunofluorescence

Cells were fixed in 4% paraformaldehyde for 10 min, followed by permeabilization using 0.1% Triton X-100 for 5 min. Nonspecific binding was minimized by blocking with 2.5% horse serum for 30 min at RT. The samples were then incubated with an anti-HCMV pp28 primary antibody (1:500) at 4°C overnight. Following incubation with primary antibody, cells were treated with TRITC-labeled goat anti-mouse IgG (1:200) for 1 h. Nuclear staining was performed with DAPI for 5 min before mounting. Fluorescent images were obtained using an MSHOT MF52-N fluorescence microscope.

### Assessment of Integrin β3 binding to HCMV glycoprotein H (gH) by ELISA

A modified ELISA assay was carried out as described in the previously published study [19]. Nickel-coated 96-well plates (Thermo Fisher) were brought to room temperature before use, followed by blocking with 2% BSA prepared in washing buffer (0.05% Tween-20 in Tris-buffered saline). His-tagged integrin β3 protein (0.1 μg per well), subjected to the indicated treatments, was added in a volume of 100 μL and incubated at 37°C for 1 h. Recombinant HCMV gH (15.6–500 ng/mL; CUSABIO Technology, Cat#CSB-YP319015HWV, Wuhan, China) was then applied to each well (100 μL) and allowed to bind for another 1 h at 37°C. The wells were then sequentially incubated with anti-HCMV gH primary antibody (1:10,000 dilution) (ViroStat, Cat#0861, Westbrook, USA) and HRP-conjugated anti-mouse IgG secondary antibody, each for 1 h at 37°C. After color development using tetramethylbenzidine (TMB) substrate, the reaction was terminated, and absorbance was measured at 450 nm using a microplate reader after gentle mixing.

### Co-immunoprecipitation analysis of Integrin β3-HCMV gH interaction

Co-immunoprecipitation (co-IP) was performed using the Immunoprecipitation Kit with Protein A+G Agarose Gel (Beyotime, Cat#P2197M, Shanghai, China) following the manufacturer’s protocol. Briefly, Cells in each group were exposed to HCMV at an MOI of 5 and incubated at 4°C for 30 min. To stabilize membrane-associated protein interactions, cells were subsequently treated with 2 mM DTSSP at 4°C for another 2 h. Cells were gently scraped and lysed in a non-denaturing lysis buffer with a protease inhibitor mixture. Integrin β3 antibodies were incubated with lysates overnight at 4°C, followed by the addition of Protein A/G Agarose beads for 2 h at 4°C. Beads were collected by centrifugation, washed with lysis buffer, and resuspended in SDS loading buffer. Proteins were separated on SDS-PAGE, transferred to PVDF membranes, and probed with HCMV gH antibodies.

### Western blot for Integrin β3-mediated HCMV entry signaling

Total cell lysates from each group were collected from 6-well plates at 37°C for 15 min post-infection (MOI = 1), washed three times with PBS, and lysed with cell lysis buffer containing a protease inhibitor cocktail. Protein concentration was determined, and 20 μg of total protein was loaded onto 10% acrylamide gels and electrophoresed at 120 V for 90 min. Proteins were transferred to polyvinylidene fluoride (PVDF) membranes, incubated overnight with specific primary antibodies, and then incubated with HRP-conjugated secondary antibodies. Detection was performed using enhanced chemiluminescence (ECL), and band intensities were quantified using ImageJ software.

### RhoA activity assay

Total cell lysates from each group were collected from 6-well plates at 37°C, 15 min after infection (MOI = 1). Intracellular GTP-RhoA activity was determined using the G-LISA® RhoA Activation Assay Biochem Kit (Cytoskeleton Inc., Cat#BK124, Denver, USA) according to the manufacturer’s instructions. Lysates were adjusted to 0.5 mg/mL using ice-cold lysis buffer. Twenty-five microliters of lysate were mixed with an equal volume of binding buffer and added to the wells of a Rho plate, followed by incubation with shaking at 4°C for 30 min. Antigen-presenting buffer was then added to each well and incubated at RT for 2 min. After washing, anti-RhoA primary antibody (1:250) and HRP-labeled secondary antibody (1:62.5) were added sequentially and incubated at RT for 45 min. HRP detection reagent was then added, and absorbance was measured at 490 nm using a BioTek microplate spectrophotometer.

### Cytoskeleton rearrangement detection

Each experimental group was infected with HCMV (MOI = 1) at 37°C for 15 min, followed by fixation in 4% paraformaldehyde at RT for 20 min. HCMV was detected by anti-pp28 antibody staining, and F-actin was stained with FITC-labeled phalloidin (ABclonal, Cat#RM02836, Wuhan, China) working solution (1:100 dilution) for 20 min in the dark [18,19]. Nuclear staining was performed using DAPI, and stress fiber morphology was observed under a fluorescence microscope.

### Statistical analysis

All data are reported as mean ± standard deviation (SD). One-way analysis of variance (ANOVA) followed by Tukey’s post hoc test was used to assess between-group differences, with adjustment for multiple comparisons using the Studentized range distribution to control the family-wise error rate at 0.05. All statistical analyses were performed using SPSS Statistics 22 (IBM, USA).

## Results

### Glycan structures and relative abundance of Integrin β3 N- and O-glycans

**Figure 1.**
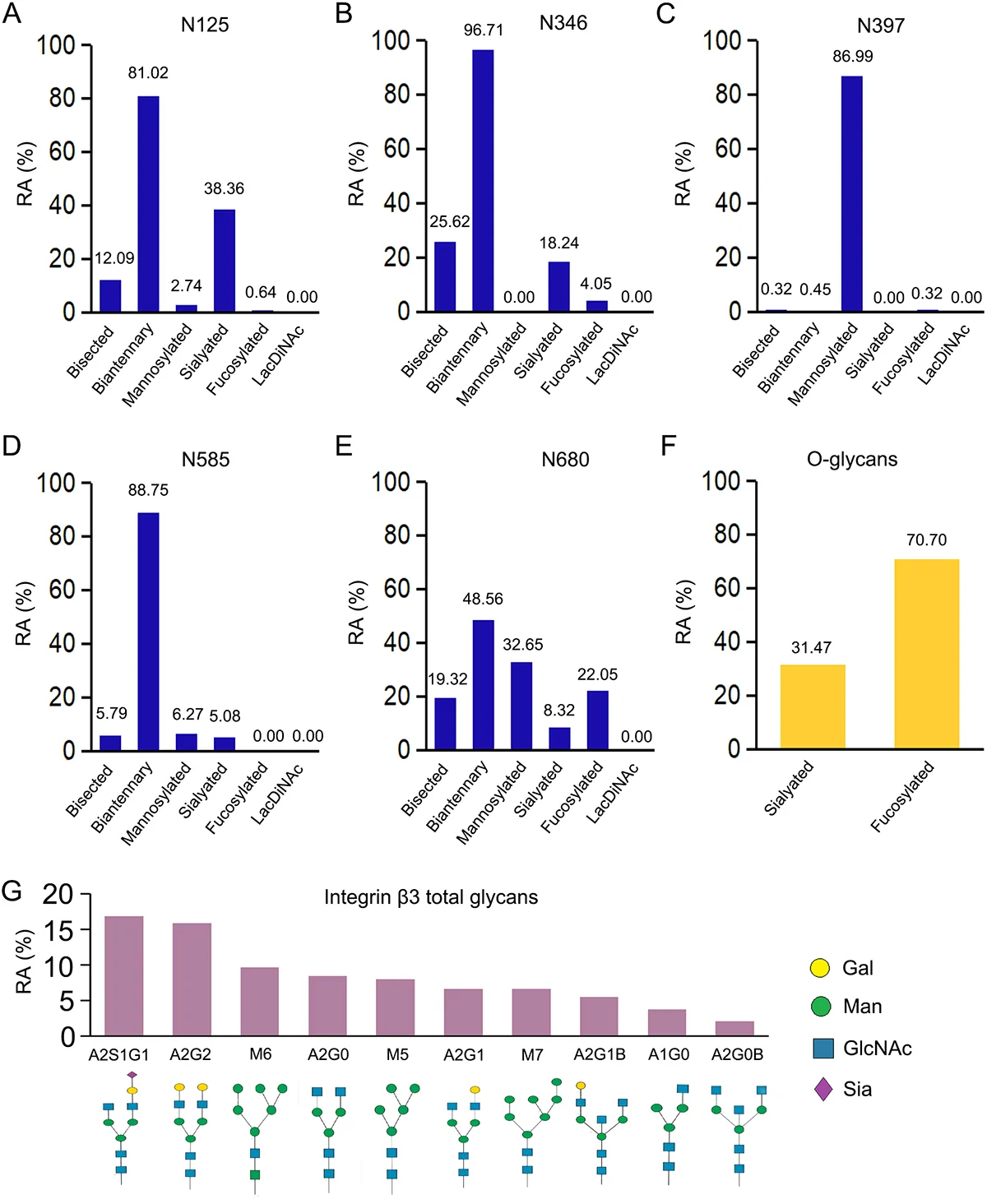
Glycan structures and relative abundance of Integrin β3. (A-E) Structures of Integrin β3 N-glycans at N125, N346, N397, N585, and N680, grouped based on structural features and relative abundance determined by high-resolution LC-MS/MS. (F) Relative abundance of sialylated and fucosylated glycans in total O-glycosylation of Integrin β3. (G) Relative abundance of the ten most abundant glycoforms of total Integrin β3 glycans (including N- and O-glycans).

Statistical analysis of glycan structures and their relative abundances at the N- and O-glycosylation sites of Integrin β3 was performed using Integrin β3 glycomics datasets previously deposited in the ProteomeXchange Consortium by the authors’ laboratory. Based on N-glycan branching classification, Integrin β3 N-glycans were predominantly biantennary structures. The relative abundances of these glycans at sites N125, N346, N397, N585, and N680 were 81.02%, 96.71%, 86.99%, 88.75%, and 48.56%, respectively (Figure 1(A-E)).

Further classification based on monosaccharide composition revealed that the N397 glycosite contained a high proportion of mannosylated glycans (86.99%). Furthermore, Integrin β3 N-glycans displayed varying levels of sialylation (including α2,3- or α2,6-linked sialic acids) and fucosylation. Sialylated glycans were most prominent at the N125 site and showed variable distribution across most glycosites except N397. Fucosylated glycans were mainly detected at the N680 site, where they constituted 22.05%, and were present at low levels at other N-glycan sites. No LacDiNAc glycans were identified among the analyzed N-glycan structures (Figure 1(A-E)).

Analysis of O-glycans indicated that sialylated O-glycans represented 31.47%, whereas fucosylated O-glycans accounted for 70.70% (Figure 1(F)). Comprehensive analysis of overall glycan abundance revealed that A2S1G1 was the most abundant glycan species, accounting for 16.8% of total glycans and derived exclusively from N-glycans (Figure 1(G)).

### Reduced Integrin β3 α2,6-sialylation resulted in an impairment in HCMV entry

To investigate the role of Integrin β3 α2,6-sialylation in HCMV entry, *ST6Gal1* knockdown and rescue assays were performed. Integrin β3 KO MRC-5 cells were produced to eliminate potential interference from other α2,6-sialylation intracellular proteins. Western blot analysis confirmed successful knockout of the Integrin β3 gene (*ITGB3*), as evidenced by the absence of Integrin β3 protein in the β3 KO group (Figure S1).

*ST6Gal1* knockdown and re-expression were subsequently verified by RT-PCR and Western blot. Both mRNA and protein levels of ST6Gal1 were significantly reduced following *ST6Gal1* gene knockdown, and were restored upon ST6Gal1 cDNA expression (Figure S2(B,C)). Lectin blot analysis further showed a marked reduction in intracellular α2,6-sialylated proteins recognized by SNA following ST6Gal1 knockdown (Figure S2(D)). Similarly, SNA lectin analysis showed that α2,6-sialylation of Integrin β3 was significantly reduced in ST6Gal1-silenced cells and restored following ST6Gal1 cDNA expression (Figure S2(E)).

**Figure 2.**
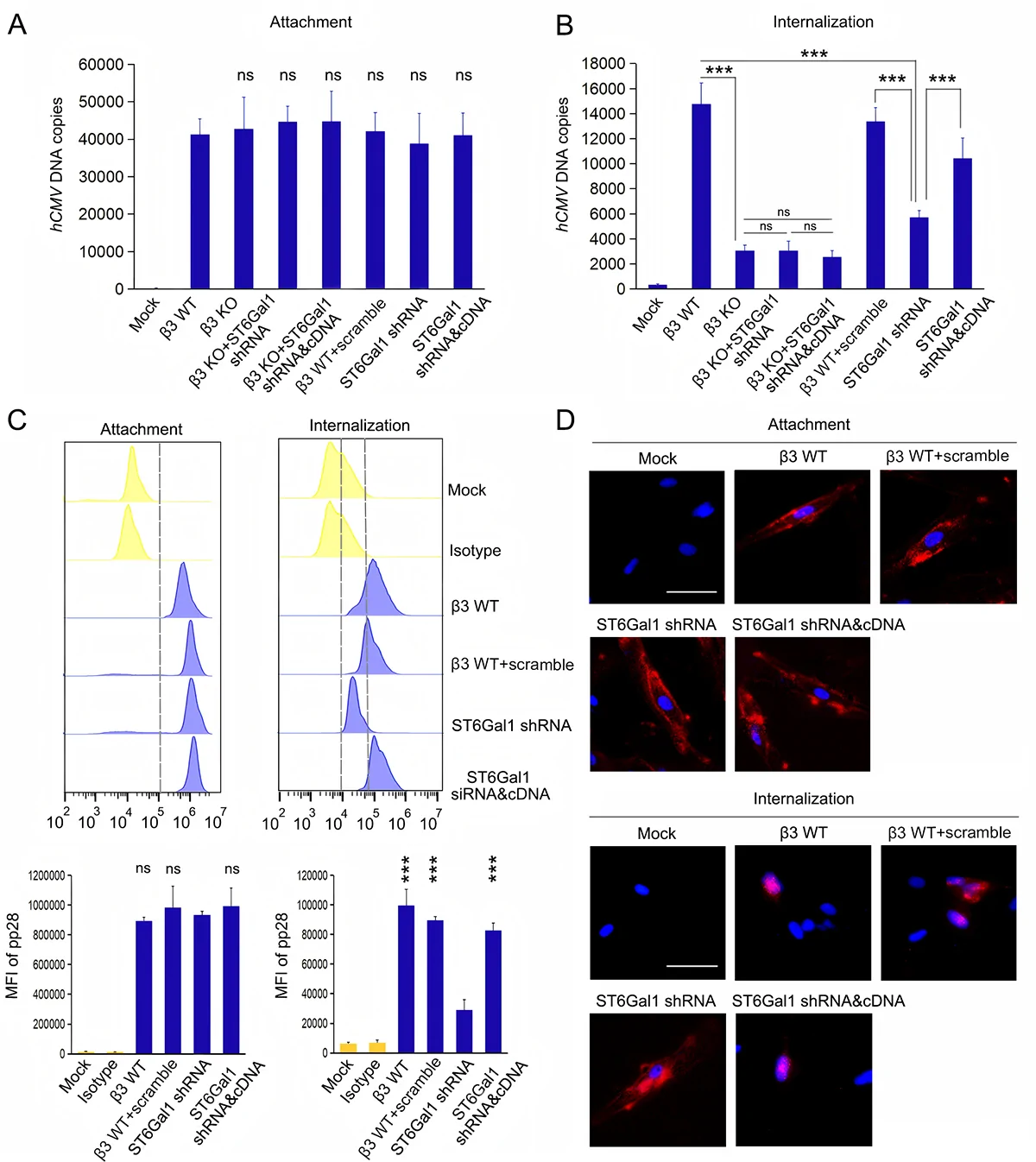
Integrin β3 sialylation defect suppresses HCMV internalization but not attachment in fibroblasts. (A) qPCR quantification of HCMV DNA copies was performed to assess viral attachment. Error bars represent standard deviations from three biological replicates. ns, not significant vs The β3 wt group. (B) qPCR quantification of HCMV DNA copies was performed to assess viral internalization. Error bars represent standard deviations from three biological replicates.***adjusted *p* < 0.001, ns, not significant. (C) Flow cytometry analysis was conducted to evaluate HCMV (MOI = 5) attachment and internalization, with at least 10,000 cells analyzed per group in three independent replicates. The corresponding MFI analysis of HCMV pp28 during viral attachment and internalization is shown in the lower panel. Data represent mean ± sd (*n* = 3). ***Adjusted *p* < 0.001 vs The ST6Gal1 shRNA group; ns, not significant vs The ST6Gal1 shRNA group. (D) Immunofluorescence imaging was performed to analyze HCMV (MOI = 50) attachment and internalization using an anti-HCMV pp28 monoclonal antibody to localize the virus. Representative images for each group are shown (magnification: 600×, scale bar: 25 μm).

Quantitative PCR was then performed to analyze HCMV attachment and internalization in fibroblasts. No significant differences in viral attachment were observed among the experimental groups (Figure 2(A)). However, Integrin β3 knockout significantly reduced intracellular viral DNA levels, indicating impaired viral internalization (Figure 2(B)). Viral DNA levels remained low in β3 KO cells regardless of *ST6Gal1*. *ST6Gal1* knockdown in wild-type (WT) Integrin β3-expressing fibroblasts significantly decreased viral internalization, whereas ST6Gal1 re-expression restored intracellular viral DNA levels. These results indicate that Integrin β3 α2,6-sialylation is required for efficient HCMV internalization (Figure 2(B)).

Flow cytometry further confirmed these findings. Comparable levels of viral attachment were detected at 4°C across all experimental groups (Figure 2, left panel). Following temperature shift to 37°C, the ST6Gal1 shRNA group displayed reduced fluorescence intensity, indicating defective viral entry (Figure 2, right panel). Immunofluorescence analysis produced consistent results, revealing surface-localized viral particles in ST6Gal1-silenced cells when incubated at 4°C (Figure 2, upper panel), whereas intracellular perinuclear localization was observed in control groups after incubation at 37°C (Figure 2, lower panel).

### Reduced Integrin β3 α2,6-sialylation resulted in a decrease in HCMV infection

**Figure 3.**
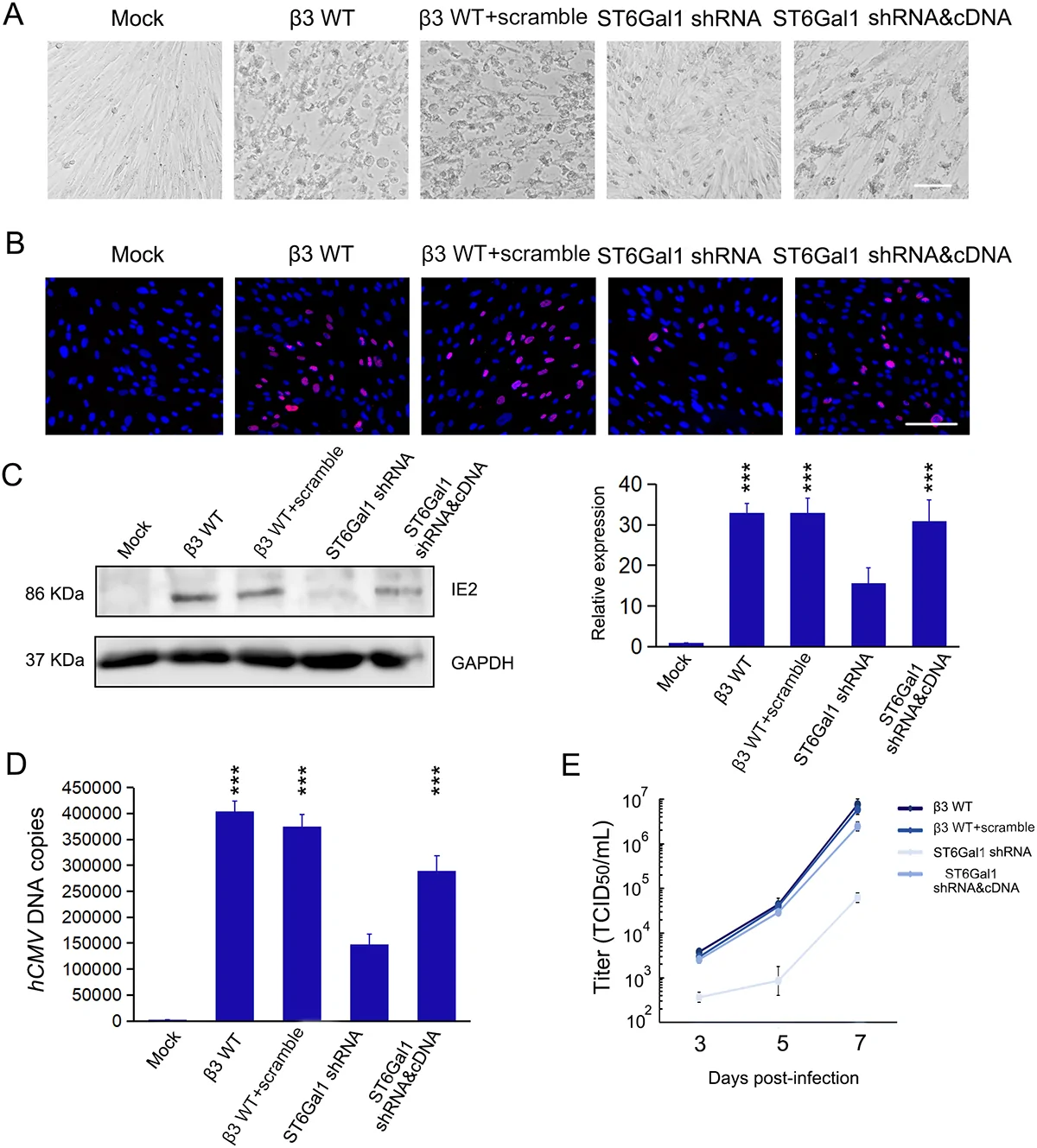
Integrin β3 sialylation defect reduces HCMV infection. (A) Cell morphology and HCMV-induced CPE were observed under a microscope at 72 hpi (magnification: 100×, scale bar: 100 μm). (B) An immunofluorescence assay was performed to assess HCMV ie protein expression across different groups (magnification: 200×, scale bar: 100 μm). (C) Western blot analysis was conducted to detect HCMV IE2 protein expression in each group. (D) Total intracellular DNA was extracted at 48 hpi, and HCMV DNA copy numbers in each indicated group were quantified by qPCR. (E) Extracellular culture supernatants were collected at 3, 5, and 7 dpi, and TCID_50_ assays were performed to measure viral titers in each group. Data are presented as mean ± standard deviation (*n* = 3). ***Adjusted *p* < 0.001 vs The ST6Gal1 shRNA group.

To further evaluate the role of Integrin β3 α2,6-sialylation in HCMV entry, several infection-associated parameters were investigated, including HCMV-induced cytopathic effects (CPE), immediate-early (IE) protein expression, viral replication, and infectious viral titers (TCID_50_). At 72 hpi, severe CPE was observed in the β3 WT and β3 WT + ST6Gal1 scramble shRNA groups, whereas the ST6Gal1 shRNA group showed only mild CPE compared with the Mock control group (Figure 3(A)). Immunofluorescence and Western blot analyses demonstrated significantly reduced IE protein levels in the *ST6Gal1* knockdown group compared with the β3 WT, scramble, and *ST6Gal1* expression rescue groups (Figure 3(B,C)). Quantitative PCR analysis showed a substantial decrease in viral DNA copy numbers in the ST6Gal1 shRNA group at 48 hpi (Figure 3). Furthermore, TCID_50_ assays revealed significantly lower infectious virion production in the ST6Gal1 shRNA group at 3, 5, and 7 days post-infection compared with the other groups. At 7 dpi, the viral titer was markedly lower in the ST6Gal1 shRNA group (TCID_50_/mL = 6.22 × 10^4^) than in the β3 WT (TCID_50_/mL = 7.60 × 10^6^) and *ST6Gal1* expression rescue groups (TCID_50_/mL = 2.45 × 10^6^) (Figure 3(E)).

### Reduced Integrin β3 a2,6-sialylation decreases the interaction between Integrin β3 and HCMV gH

**Figure 4.**
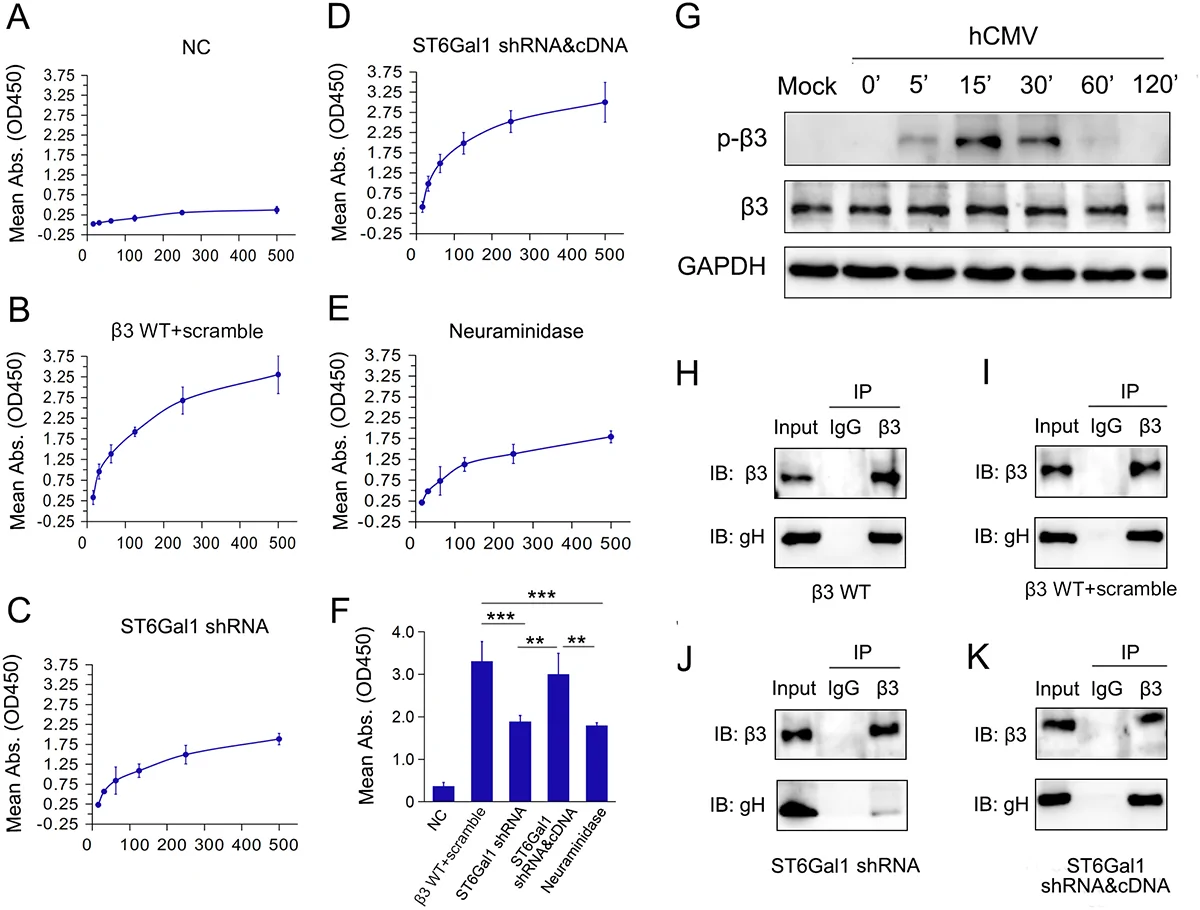
Integrin β3 sialylation defect reduces the interaction between Integrin β3 and the HCMV ligand gH. (A-F) ELISA analysis demonstrated altered binding of serially diluted HCMV ligand gH to recombinant Integrin β3 with *ST6Gal1* knockdown or neuraminidase treatment. The X-axis represents the concentration of recombinant gH (ng/mL). **Adjusted *p* < 0.01, ***adjusted *p* < 0.001. (G) Western blot analysis was performed to investigate Integrin β3 activation at different time points post-infection. (H–K) Co-IP assays were conducted to assess the interaction between HCMV gH and Integrin β3, with and without *ST6Gal1* knockdown, during HCMV entry.

To clarify the molecular mechanisms underlying Integrin β3-mediated HCMV internalization, the interaction between Integrin β3 and the HCMV glycoprotein gH was investigated. Recombinant wild-type Integrin β3, with or without ST6Gal1 shRNA co-transfection, was expressed and purified from HEK 293T cells, and its binding to HCMV gH was assessed using a modified ELISA assay. The results demonstrated that *ST6Gal1* knockdown significantly reduced the binding between Integrin β3 and HCMV gH compared with the scramble control and ST6Gal1 rescue group (Figure 4(A-D) and 4(F)). Neuraminidase digestion of Integrin β3 was performed to further evaluate the contribution of sialic acids. Western blot analysis showed a reduction in the molecular weight of Integrin β3, while lectin blot analysis confirmed a marked reduction in sialic acids on Integrin β3 following neuraminidase treatment (Figure S3A,B). Removal of surface sialic acids significantly decreased Integrin binding to gH (Figure 4(E, F)).

Kinetic analysis indicated that Integrin β3 activation peaked approximately 30 min after HCMV infection (Figure 4(G)). Co-immunoprecipitation assays further demonstrated strong interactions between Integrin β3 and HCMV gH in β3 WT, scramble, and ST6Gal1 shRNA and cDNA groups (Figure 4(H, I) and 4(K)). *ST6Gal1* knockdown significantly reduced this interaction (Figure 4(J)). These findings indicate that Integrin β3 α2,6-sialylation is critical for its interaction with HCMV gH.

### Integrin β3 a2,6-sialylation mediates HCMV entry signaling

**Figure 5.**
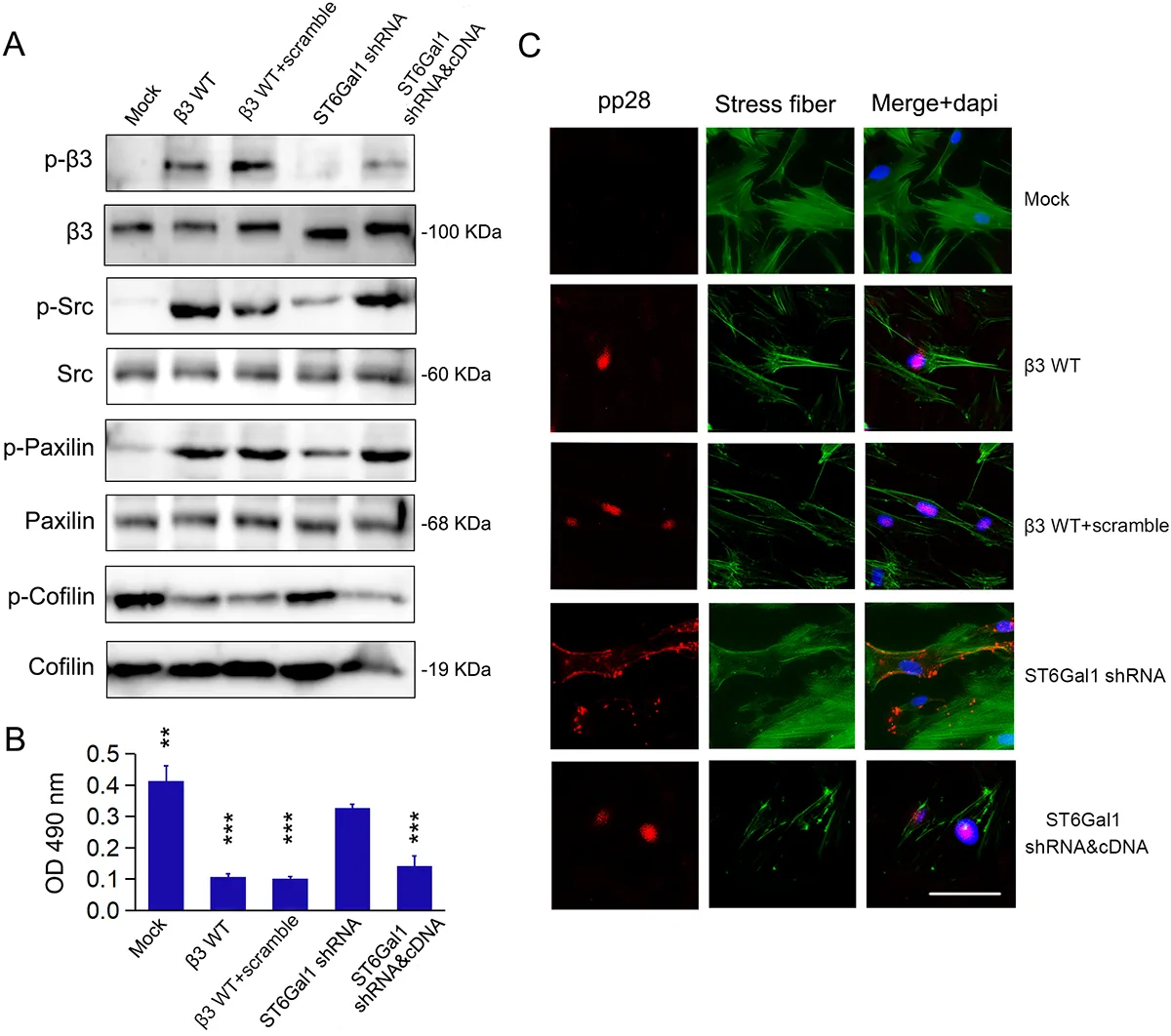
Integrin β3 sialylation participates in HCMV-induced cytoskeleton rearrangement during viral entry. (A) Integrin β3 sialylation defect resulted in downregulation of the Integrin β3-src signaling pathway. (B) The sialylation defect also resulted in impaired HCMV-induced RhoA inactivation, as shown by quantification. Data are presented as mean ± standard deviation (*n* = 3), with **adjusted *p* < 0.01 vs The ST6Gal1 shRNA group, ***adjusted *p* < 0.001 vs The ST6Gal1 shRNA group. (C) Integrin β3 S85 sialylation was found to facilitate stress fiber disassembly and promote viral nuclear translocation. Representative immunofluorescence images for each group are shown (magnification: 600×, scale bar: 25 μm).

HCMV receptor-ligand interactions activate the Integrin/Src and RhoA/cofilin pathways, which orchestrate actin stress fiber rearrangements necessary for viral internalization. ST6Gal1 gene knockdown resulted in decreased α2,6-sialylation of Integrin β3, as indicated by a reduction in its molecular weight (Figure 5(A)). Downstream activation of Src and Paxillin was suppressed (Figure 5(A)). β3 WT, scramble, and ST6Gal1 shRNA and cDNA groups displayed robust β3/Src pathway activation upon HCMV infection. The RhoA/cofilin pathway showed high levels of GTP-RhoA (Figure 5(B)) and cofilin phosphorylation in ST6Gal1 shRNA and Mock groups, indicating cytoskeletal stabilization (Figure 5(A)).

Fluorescence imaging of F-actin at 1 hpi revealed significant disassembly of actin stress fibers and cell contraction in β3 WT, scramble, and ST6Gal1 shRNA and cDNA groups, along with perinuclear localization of pp28, consistent with successful viral internalization (Figure 5). In contrast, ST6Gal1 knockdown cells retained intact stress fibers, and pp298 remained restricted to the cell surface (Figure 5). These results underscore the critical role of Integrin β3 α2,6-sialylation in regulating HCMV entry signaling and cytoskeleton remodeling.

## Discussion

This study investigated Integrin β3 sialylation to dissect its role in HCMV entry into fibroblasts. In mammals, sialic acids are typically located at glycan termini, forming α2,6-, α2,3-, and α2,8-linkage [24,25]. Glycopeptide analysis of Integrin β3 revealed that A2S1G1, containing terminal SAs, was the most abundant glycan. Except for the highly mannosylated N397 site, sialylated glycans were broadly distributed across Integrin β3. The prevalence of terminal SAs supports their functional importance in viral entry and justifies the focus on Integrin β3 sialylation. This study extends previous work by the authors demonstrating the involvement of specific N- and O-glycosylation sites on Integrin β3 in HCMV entry. It represents a follow-up investigation into the role of specific glycan structures in viral entry, providing a comprehensive understanding of how Integrin β3 glycosylation mediates HCMV infection in fibroblasts.

Previous work identified N125 and S85 as critical glycosylation sites on Integrin β3 for HCMV internalization [19,20]. Glycan profiling revealed that A2S1G1, the most abundant N125 glycan, represents the predominant glycoform across Integrin β3 [19]. This glycan is enriched in terminal sialic acids and likely contains predominantly α2,6-sialylation linkages, consistent with its role in promoting viral internalization. The N125 and S85 are positioned near the Integrin β I-like domain, which contains ligand-binding motifs and mediates signaling pathways [26,27]. The spatial arrangements and structural characteristics of these glycosites may influence I-like domain conformation, enhancing the interaction between Integrin β3 and HCMV ligands. O-glycans at other sites display low glycosylation efficiency (0.1% to 2.2%) except at S85 [20], suggesting minimal contribution to viral entry.

Host glycosylation has been shown to play an important role in herpesvirus–host interactions. Our observation that Integrin β3 glycosylation mediates HCMV entry is supported by findings from other herpesviruses, including *Herpes simplex* virus 1 (HSV-1), in which host glycosylation has been implicated in viral infection. 3-O-sulfated heparan sulfate, a cell-surface glycosylation modification, has been reported to interact with HSV-1 glycoprotein D (gD), contributing to initial viral entry and membrane fusion [28,29]. These observations raise the possibility that interactions with host surface glycosylation or specific glycans represent a common mechanism underlying herpesvirus infection.

The early stages of viral infection are often mediated by SAs on host or viral surfaces. Coronaviruses (CoVs) utilize SAs and specific linkages for host cell entry. It has been reported that transmissible gastroenteritis coronavirus (TGEV) interacts with SA species on mucin-like glycoproteins (MGPs) to traverse the mucus barrier and infect intestinal enterocytes [30,31]. The γ-CoVs, infectious bronchitis virus (IBV), preferentially bind 2,3-linked NeuAc on tracheal and lung epithelial cells [32], with the SA-binding site located on the S1 subunit of the spike protein, while the protein receptor remains unknown. Orthoreovirus sigma 1 engages SAs before interacting with junctional adhesion molecule 1 (JAM-1), a secondary viral receptor essential for endocytosis [33]. Merkel cell polyomavirus (MCPyV) binds SAs, inducing conformational changes that enable Integrin α4β1 engagement, and SA interaction with gangliosides can promote caveolin-mediated endocytosis [34,35]. The current findings demonstrate that α2,6-linked SAs on Integrin β3 are required for HCMV internalization. Furthermore, ST6Gal1 knockdown may induce compensatory intracellular glycosylation mechanisms, including increased α2,3-sialylation mediated by ST3Gal enzymes. Although compensatory α2,3-sialylation may occur, our results demonstrate that it remains insufficient to reverse the marked reduction in HCMV entry. This implies that HCMV preferentially binds α2,6-linked SAs.

This study has several limitations. First, only the involvement of Integrin β3 α2,6-sialylation in the entry of HCMV Towne strain into fibroblasts was investigated, without assessing other strains, including AD169 or clinical isolates. Second, due to methodological constraints, α2,6-sialic acids could not be selectively deleted or added at the N125 glycan terminal of Integrin β3. Inferences regarding the role of N125 α2,6-sialylation rely on combined results from this study and previous N-glycosite mutation analyses, indicating its central role in viral entry. Third, the broad tropism of HCMV implies that the glycosylation of other receptors in endothelial cells and macrophages during early infection should be further investigated. Given the widespread presence of cellular α2,6-linked sialic acid modifications, it is hypothesized that HCMV receptors on endothelial and epithelial cells, such as Neuropilin-2, may also be sialylated and thereby contribute to HCMV entry. This possibility warrants further investigation. Furthermore, the potential involvement of glycans on HCMV envelope glycoproteins in host cell entry warrants investigation. Despite these limitations, the results show that Integrin β3 α2,6-sialylation facilitates HCMV internalization in fibroblasts, highlighting Integrin β3 sialylation and ST6Gal1 as potential targets for early-stage HCMV intervention.

## Supplementary Material

Supply materials.docx

## Data Availability

The data is publicly available on the findings of this study and unrestricted re-use is permitted via an open license. The data generated during the study is available at https://doi.org/10.6084/m9.figshare.31908340 [36].
