# Supplementary material for: Integrin β3 α2,6-sialylation by the sialyltransferase ST6Gal1 promotes human cytomegalovirus entry into fibroblasts
Source: Virulence. 2026 Sep 11;17(1):2732519. doi: 10.1080/21505594.2026.2732519 (PMC13577289; doi:10.1080/21505594.2026.2732519)
Supplement: Supplementary file 1 [file KVIR_A_2732519_SM2794.docx]

**Table S1. shRNA sequences**

| **Gene names** | **shRNA1 (5’-3’)** | **shRNA2 (5’-3’)** |
| --- | --- | --- |
| ***ST6Gal1*** | CUCUCAGUUGGUUACCACA | CUGGGAUGCUUGGUAUCAU |

**Table S2. Primer sequences for RT-PCR of the *ST6Gal1* and *GAPDH* genes**

| **Gene names** | **Sense (5’-3’)** | **Antisense (5’-3’)** |
| --- | --- | --- |
| ***ST6Gal1*** | GAAAAATGGGCCTTGGCCTG | GAGCAGGAAAACAAGCCTGC |
| ***GAPDH*** | GCACCGTCAAGGCTGAGAAC | TGGTGAAGACGCCAGTGGA |

**Table S3. Primer sequences for *qPCR* of the HCMV *UL123* gene**

| **Gene names** | **Sense (5’-3’)** | **Antisense (5’-3’)** |
| --- | --- | --- |
| **HCMV *UL123*** | **TCTGCCAGGACATCTTTCTC** | **GTGACCAAGGCCACGACGTT** |

**Figure S1. Validation of Integrin β3 knockout.** Western blot was performed to confirm Integrin β3 knockout in MRC-5 cells.

**Figure S2. Validation of ST6Gal1 expression.** ST6Gal1 expression was evaluated by RT-PCR (A-B) and Western blotting (C) in the indicated cell groups. (D) α2,6-sialylation levels of total cellular proteins in each group were evaluated by SNA lectin blot. (E) Cellular Integrin β3 from each group was immunoprecipitated, and its α2,6-sialylation levels were evaluated by SNA lectin blot. CBB: Coomassie Brilliant Blue.

**Figure S3. Verification of recombinant Integrin β3 de-glycosylation after glycosidase treatment.** (A) Representative Western blot showing recombinant Integrin β3 protein pretreated with neuraminidase. The de-glycosylation effect was confirmed by differences in electrophoretic migration. (B) Sialylation levels of recombinant Integrin β3 with or without neuraminidase treatment were evaluated by SNA lectin blot. CBB: Coomassie Brilliant Blue.
